# Sperm Chromatin Condensation Defect Accelerates the Kinetics of Early Embryonic Development but Does Not Modify ICSI Outcome

**DOI:** 10.3390/ijms24010393

**Published:** 2022-12-26

**Authors:** Fanny Jumeau, Nathalie Rives, Pauline Lechevallier, Coline Boniou, Maria Letailleur, Audrey Réal-Lhommet, Aurélie Feraille

**Affiliations:** 1 Team Adrenal and Gonadal Pathophysiology, Reproductive Biology Laboratory—CECOS, Rouen University Hospital, University Rouen Normandie, Inserm, U1239 NorDIC, F-76000 Rouen, France; 2 Assisted Reproductive Center, Department of Gynaecology and Obstetrics, Rouen Normandy University Hospital, F-76000 Rouen, France

**Keywords:** embryo morphokinetics, ICSI outcomes, paternal contribution, sperm condensation, sperm DNA fragmentation, time-lapse

## Abstract

The origin and quality of gametes are likely to influence the kinetics of embryonic development. The purpose of the study was to assess the impact of sperm nuclear quality, and in particular sperm chromatin condensation, on the kinetics of early embryo development after intracytoplasmic sperm injection (ICSI). Our study included 157 couples who benefitted from ICSI for male factor infertility. Chromatin condensation and DNA fragmentation were assessed in spermatozoa prior to ICSI. Above the 20% threshold of sperm condensation defect, patients were included in the abnormal sperm chromatin condensation (ASCC) group; below the 20% threshold, patients were included in the normal sperm chromatin condensation (NSCC) group. After ICSI, the oocytes were placed in the time-lapse incubator. The kinetics of the cohort’s embryonic development have been modeled. The fading times of pronuclei and the time to two blastomeres (t2, first cleavage) and four blastomeres (t4, third cleavage) differed significantly between the NSCC and ASCC groups, with earlier events occurring in the ASCC group. On the other hand, the state of sperm chromatin condensation did not seem to have an impact on live birth rates or the occurrence of miscarriages. The kinetics of early embryonic development was accelerated in males with a sperm chromatin condensation defect without compromising the chances of pregnancy or promoting miscarriage. However, our study highlights the paternal contribution to early embryonic events and potentially to the future health of the conceptus.

## 1. Introduction

In infertile males, sperm exploration is generally based on conventional semen parameters according to World Health Organization (WHO) criteria (concentration, motility, vitality and morphology) [1]. Sperm concentration and motility appear to be the most discriminating and predictive factors for in vitro fertilization (IVF) success [2]. Nevertheless, other semen parameters can be explored, in particular those related to sperm nuclear quality (i.e., numerical chromosome abnormalities, DNA fragmentation, chromatin condensation defect, presence of large vacuoles in the sperm head) [3,4].

Sperm nuclear quality can interfere with normal embryo development. In particular, high levels of sperm aneuploidy have been more frequently reported in cases of IVF failure [5,6]. Sperm DNA fragmentation can alter embryo development from fertilization to the blastocyst stage with a decrease in implantation rates [7]. Sperm chromatin condensation defect has been associated with absent or low embryo development after intracytoplasmic sperm injection (ICSI) [8] as well as with a decrease in pregnancy rate and was more frequently observed in couples with ICSI failure [9,10]. Sperm chromatin condensation defect has been proposed as a negative predictive factor for the occurrence of clinical pregnancy after ICSI [4].

Studies exploring the paternal role and specifically the influence of sperm parameters on the kinetics of embryonic development led to contradictory data and included a low number of patients. Male factor infertility could lead to fewer usable embryos but did not modify the overall time to the blastocyst stage. Sperm characteristics able to alter embryo development might not be the same as those assessed during routine semen analysis [11]. For example, severe sperm head morphology alterations observed during intracytoplasmic morphologically selected sperm injection (IMSI) did not seem to impact embryo morphokinetic parameters [12]. However, the morphokinetic parameters appeared to be disturbed according to the morphological type of microinjected spermatozoa [13]. In addition, a positive correlation has been detected between sperm DNA fragmentation rate and the kinetics of embryo development up to the blastocyst stage [14]. The presence of sperm nuclear abnormalities (i.e., DNA fragmentation, chromatin condensation defect) disturbed the division to two (t2) and three (t3) blastomeres. However, the predictive character of one particular sperm nuclear abnormality was not demonstrated and the kinetics of early embryo development was not modeled [15].

Since the implementation of the time-lapse system, studies have focused on the development of algorithms, by retrospective comparison of kinetics leading or not to a pregnancy or a live birth, allowing an embryo with high implantation potential to be selected or conversely an embryo with poor prognosis to be identified [16,17,18]. The evaluation of these different algorithms has highlighted that a single decision tree algorithm is not very helpful and that it is also necessary to take into account the clinical and biological context of the infertile couple as well as the embryo culture conditions [19].

In light of these findings, sperm nuclear quality could have an impact on the kinetics of early embryonic development and could affect the birth rate after ICSI. The aim of the current study was to evaluate the impact of sperm nuclear quality and, in particular, chromatin condensation and DNA fragmentation on the kinetics of early embryo development after ICSI and to expand the range of potential paternal parameters as non-invasive biomarkers of ICSI outcome.

## 2. Results

### 2.1. Characteristics of the Study Population

The demographic, clinical and conventional semen characteristics and sperm nuclear damages are reported in Table 1, and the ICSI cycle parameters are presented in Table 2. The ICSI parameter attempts are expressed per couple or cycle. Indeed, a total of 157 couples who benefitted from 157 ICSI cycles were included in the study, leading to the analysis of morphologic and early morphokinetic parameters of 681 embryos, 74 biochemical pregnancies, 62 clinical pregnancies and 57 live births of healthy children. Close to 14% (13.4%, 21/157) of infertile males presented an increased sperm chromatin condensation defect rate (26.2 ± 4.7%).

First, we compared the characteristics of the two groups of infertile couples/ICSI cycles resulting in a live birth or not (Table 1 and Table 2). No significant differences were found between the “live birth” and “no live birth” groups regarding male and female parameters (age, duration of infertility, antral follicle count (AFC), follicle-stimulating hormone (FSH), luteinizing hormone (LH), estradiol (E_2_)_,_ conventional semen parameters and sperm nuclear damage) and ICSI cycles (mean rank of ICSI cycle, E_2_ at triggering, number of oocytes collected and injected, fertilization rate, number of embryos obtained and frozen). However, the female body mass index (BMI) was significantly increased in the “no live birth” group. In the “live birth” group, anti-Müllerian hormone (AMH) values and the number of embryos transferred were significantly higher, while the total doses of FSH administered were lower in the “no live birth” group. Second, we compared the same characteristics as mentioned above considering the normalcy or not of the sperm chromatin condensation state. Despite the statistically significant impact of female partner (BMI, AMH, total FSH administered doses) as well as the number of embryo transfers on live birth rate, when adjusting for sperm condensation status, these parameters were no longer significant (Table 1 and Table 2). However, sperm progressive motility was significantly lower in the ASCC group compared to the NSCC group. The chromatin condensation rate was significantly higher in the ASCC group compared to the NSCC group. The other semen parameters, sperm DNA fragmentation and the pregnancy outcomes did not differ significantly between the two groups.

### 2.2. Impact of the Sperm Chromatin Condensation State on the Embryo Morphology and Early Morphokinetic Parameters

The morphology and morphokinetic parameters of early embryo development are presented in Table 3. No correlation was found between the morphokinetic parameters and the chromatin condensation state. No significant differences were found regarding the timing of expulsion of the second polar cell (tPB2), the appearance of *pronuclei* (tPNa) and the time to three blastomeres (t3) between the ASCC and NSCC groups.

The fading times of *pronuclei* (tPNf) and the time to two (t2) and four (t4) blastomeres were significantly different between the ASCC and NSCC groups, with earlier events occurring in the ASCC group. The S-phase and the first cell cycle (ECCC1) were significantly shorter in the ASCC group compared to the NSCC group. However, the duration of the second cell cycle (ECCC2) and the synchronization phase of the cell divisions (S2) were not different between the two groups.

## 3. Discussion

This study explored the impact of sperm chromatin condensation defects on the first stages of embryonic kinetics in couples managed with ICSI. Our data showed an acceleration of the kinetics of the early embryonic events when infertile males presented a higher sperm chromatin condensation defect rate. Indeed, reductions in the time of *pronuclei* fading (tPNf), the time to two (t2) and four (t4) blastomeres and the S-phase length were observed. Our study highlights the paternal contribution to early embryonic events and potentially to the future health of the conceptus even if the state of sperm chromatin condensation did not seem to have an impact on the live birth rates or the occurrence of miscarriages.

The infertile males included in our study have oligozoospermia alone or associated with asthenozoospermia. The sperm count was comparable in both groups. However, the ASCC group showed a significantly decreased progressive motility compared to the NSCC group. Indeed, abnormal chromatin remodeling during spermiogenesis due to inappropriate protamination (histone to protamine replacement) can alter sperm head size into an optimal configuration for motility [20]. To our knowledge, no studies have shown a relationship between sperm progressive motility and the kinetics of early embryonic development even if a positive correlation has been reported between non-progressive motility and t2, t3 and t4 [15].

In our study, the sperm chromatin condensation state did not influence the fertilization rates. It has been proposed that sperm with abnormal chromatin condensation can fertilize an oocyte after ICSI and transmit DNA damage into the fertilized oocyte and consequently into the developing embryo, often leading to impaired embryonic development and miscarriage [21]. Indeed, the kinetics of embryonic development was disrupted in our study and, in particular, the first cell cycle. The fading of p*ronuclei* occurred significantly earlier in the ASCC group compared to the NSCC group. This event was found in a non-significant way among embryos that failed to implant compared to those that led to implantation [22,23]. However, these data are controversial because other studies have shown that early deletion of *pronuclei* is associated with a better implantation rate [24,25] and live birth rate [26].

An accelerated kinetics of early embryonic development has also been observed in ICSI-derived embryos using surgically retrieved testicular spermatozoa. Testicular spermatozoa have immature chromatin compared to ejaculated sperm cells; i.e., they do not carry out the complete nuclear modifications observed during the epididymal transit, such as increased adding of disulfide bridges between cysteine residues of protamine molecules to produce a tightly condensed chromatin structure [27]. The abnormal chromatin condensation observed in ejaculated spermatozoa of our patients and the immaturity of testicular sperm chromatin could have the same consequences on the kinetics of early embryonic development. The absence of or failure to set up disulfide bridges could allow faster access to the mechanisms for remodeling genetic material after fertilization and thus could have an impact on the kinetics of early embryonic events. Early embryonic events correspond to the entry of the embryo into the first phase, M, i.e., the step of mixing male and female genetic material which requires a complete decondensation of the genome [28]. In addition, during the first 24 h of embryonic development before the initiation of the S-phase, DNA damage repair (DDR) is increased in the zygote in order to repair damage in both parental genomes (sperm and oocyte nuclei) [29]. Dysregulation of DDR in sperm and oocyte nuclei or reduced time to DDR that can be considered in our study could have several consequences for the zygote and embryo: (i) decreased viability and (ii) tolerance of lesions with an increased risk of genetic or epigenetic disorders being inherited by offspring conceived by ICSI, as well as predisposition to cancer and rapid-aging diseases [30,31]. Indeed, impairment of sperm chromatin remodeling during spermiogenesis renders DNA vulnerable to DNA damage induced by oxidative stress that should be repaired by the oocyte prior to the S-phase of the first mitotic division. Any deficiency or inaccuracy in DDR has the potential to fix paternal DNA damage as a de novo mutation in the embryo and consequently in offspring [32].

The S-phase, i.e., the DNA replication step, is significantly shorter in the ASCC group compared to the NSCC group. The shorter duration of the S-phase has already been reported to be associated with a significantly lower implantation rate [24]. In our study, t2 (first cleavage), ECC1 (duration of the cell cycle) and t4 (third cleavage) occurred significantly earlier in the ASCC group compared to the NSCC group. Published data indicate that a precocious first cleavage (t2 < 25.9 h) is predictive of blastulation [33,34], embryonic quality on day 3 post-fertilization [35] and a better implantation rate [25,33]. The t4 seems to be associated with the formation of a good-quality blastocyst [33] and a better implantation rate [19,25,33] in favor of early onset. However, semen parameters were not described in these different studies. In our study, the duration of the second cell cycle (ECC2) did not seem to be affected by the state of sperm chromatin condensation and suggests the existence of potential early embryonic repair mechanisms. Few studies have investigated the paternal effects on early embryo events as compared with studies on maternal effects, notably using the time-lapse system. A recent study assessed the male contribution in early embryo development using oocytes from donors to analyze the paternal effects with a tendency for delayed kinetics in embryos from oligospermic males starting from tPB2. The authors hypothesized that the delayed timing could be the consequence of the process of DDR occurring during the first step of embryo reprogramming. However, they did not explore sperm DNA damage parameters such as sperm chromatin state [36]. 

Our study has some limitations. Our study was a retrospective analysis with a small sample subset size. We were not able to set up the characteristics of the single sperm used for ICSI cycles because we performed a conventional ICSI. Nevertheless, only progressive and motile spermatozoa with a normal form (sperm head, nucleus and flagellum) were injected into the oocytes. However, the normalcy of the sperm nucleus assessed during ICSI procedure could partly but not completely indicate the normalcy of chromatin condensation. Our study also has some strengths. Several other factors can influence the kinetics of in vitro embryo development. Indeed, the insemination technique used seems to have an impact, with earlier embryo development in ICSI compared to IVF [37]. However, all embryos were obtained after ICSI in our study. In addition, culture medium [38,39] as well as gas concentrations [40] could influence the kinetics of embryonic development. These parameters cannot explain our findings because all embryos were cultured under the same conditions. Sperm DNA damage such as DNA fragmentation can also be involved in abnormal embryonic development. Indeed, sperm DNA fragmentation could hinder and slow down early embryonic development [14,41]. However, Mangoli et al. did not find any link between sperm DNA fragmentation and the kinetics of embryonic development [15]. In our study, the rate of sperm DNA fragmentation was comparable between the ASCC and NSCC groups and could not influence the modification observed in the first stages of embryonic kinetics. In addition, sperm chromatin condensation defects did not appear to be associated with sperm DNA fragmentation. Finally, the standard semen parameters and male and female sociodemographic characteristics did not differ significantly between the two groups.

In conclusion, our findings show that the role of sperm in embryogenesis goes beyond genomic material transfer. Indeed, chromatin sperm structure can interfere with early embryonic development in case of chromatin condensation defects. Indeed, abnormal sperm chromatin compaction could allow faster access to the fusion mechanisms of the maternal and paternal genomes and explain why the stages of embryonic development were earlier in our study. The chromatin condensation defect could also reflect other epigenetic modifications such as DNA methylation defects, the impact of which on the *conceptus* remains to be assessed. Our study highlights the paternal contribution to the early embryonic events and consequently to the future health of the conceptus. Indeed, ICSI of IVF outcome is mostly expressed in a quantitative manner using clinical pregnancy or live birth rates. However, our study was focused on the qualitative aspect of ICSI or IVF outcome, considering that a sperm chromatin condensation defect, by reducing the timing of early embryo events, a major period for DNA damage repair in gametes, could alter the conceptus quality and have an impact on the mid- and long-term offspring health. According to our results, the clinical–biological context and, in particular, sperm DNA damage should also be taken into account in the analysis of early parameters of embryonic morphokinetics. The etiology of sperm chromatin condensation defects should be explored in order to improve the chromatin state before the ICSI procedure. 

## 4. Materials and Methods

This retrospective observational study was conducted at the Reproductive Biology Laboratory-CECOS of Rouen University Hospital from 1 January 2014, to 31 December 2017.

Inclusion criteria were infertile couples consulting for male factor infertility after a 12-month trial to achieve spontaneous pregnancy. The couples benefitted from ICSI regardless of the rank of the cycle (1 to 2) performed in our laboratory. Only ICSI cycles were included in order to control the time of insemination and to report fertilization-related measures obtained after the culture of fertilized oocytes in the tri-gas time-lapse incubator (Embryoscope, Unisense Fertilitech, Aarhus, Denmark). One cycle was included per couple. Infertile males presented conventional semen parameter alterations (sperm concentration (10^6^/mL), total sperm number (10^6^/ejaculate), progressive motility (WHO grades a + b combined, %) and vitality (%)) defined according to the World Health Organization criteria [1] and morphology (sperm normal morphology, %) [42]. They benefited from the assessment of sperm chromatin condensation defects and DNA fragmentation. All infertile males have normal blood karyotypes. 

Exclusion criteria were infertile males with (i) obstructive and non-obstructive azoospermia with the use of fresh or cryopreserved surgically retrieved spermatozoa, (ii) cryptozoospermia or severe oligozoospermia (sperm count less than 1 million/ejaculate), (iii) urinary spermatozoa, (iv) frozen spermatozoa (i.e., fertility preservation or sperm donation), (v) abnormal blood karyotype; ICSI performed in a viral context (active syphilis, hepatitis B or C, HIV); women above 42 years; and egg donation.

Information used in the study was collected for clinical use and was recorded from the patients’ electronic medical files. Demographic and clinical characteristics included delay of infertility (years), age (years), body mass index (BMI, kg/m^2^), antral follicle count (AFC), basal serum of luteinizing hormone (LH) (UI/L), basal serum of follicle-stimulating hormone (FSH) (UI/L), basal serum of 17β estradiol (E_2_) (pg/mL) and anti-Müllerian hormone (AMH) (ng/mL). We completed data using morphologic and morphokinetic descriptions of the embryos obtained during ICSI cycles.

All the techniques used are standard techniques in the Reproductive Biology Laboratory-CECOS of Rouen University Hospital (Rouen, France). The current study is a non-interventional study and respects the current French Bioethics Law and French Public Health Law. Informed consent was not required due to the retrospective nature of the study. Ethical approval was obtained from the Institutional Ethical Committee for Non-Interventional Research of Rouen University Hospital.

### 4.1. Conventional Semen Analysis

Sperm collection was performed after a sexual abstinence period varying between 3 and 5 days, and sperm were directly collected by masturbation into a sterile container (Clinisperm, CML, Nemours, France) at the Reproductive Biology Laboratory-CECOS. After liquefaction at room temperature, the conventional semen parameters were explored and analyzed according to WHO recommendations [1]. Sperm morphology was studied according to David’s modified classification [42]. Semen parameter alterations were confirmed using two samples collected at 3-month intervals. Sperm nuclear damages were explored in a fraction of the whole semen sample.

### 4.2. Sperm Chromatin Condensation State

A fraction of the whole semen sample (0.5 to 1 mL) was washed twice with phosphate-buffered saline (PBS, BioMérieux, Marcy l’Etoile, France) 1X after centrifugation. The pellet was spread on slides (Superfrost Plus, ThermoScientific, Waltham, MA, USA) before being fixed with glutaraldehyde 3% (Glutaraldehyde, Sigma, Saint Louis, MO, USA) for 30 min at room temperature. After fixation, slides were stained with aniline blue (AB) staining at 5% pH 3.5 for 5 min (Gurr, BDH Laboratory Supplies, Dorset, UK) before dehydration in alcohol baths (70, 90 and 100%, 1 min each), immersion in xylene and mounting in Eukitt (Eukitt, EUK 100, CML, Nemours, France). A total of 500 spermatozoa were counted under a light microscope (Leitz DMRD, Leica, Solms, Germany) according to previously published criteria [4]. The percentage of spermatozoa with abnormal chromatin condensation was calculated per patient by the ratio between the number of sperm nuclei positive for AB staining and the total number of explored spermatozoa. Infertile males with a percentage of AB-positive spermatozoa above the abnormal 20% threshold value [4,43] were included in the “abnormal sperm chromatin condensation” (ASCC) group, and patients were included in the “normal sperm chromatin condensation” (NSCC) group if this percentage was below 20%.

### 4.3. Sperm DNA Fragmentation

The sperm pellet was suspended in pure methanol (EPR Methanol, Carlo Erba Reagents, Val de Reuil, France) and fixed for 30 min at −20 °C before being spread on slides (Superfrost Plus). The terminal deoxynucleotidyl transferase dUTP nick-end labeling (TUNEL) assay (In Situ Cell Death Kit Detection POD, Roche, Mannheim, Germany) was performed according to the supplier’s recommendations and published criteria [4]. DNA fragmentation was characterized in 500 spermatozoa at ×1000 magnification using an epifluorescence microscope (DMRB, Leica, Solms, Germany). The percentage of spermatozoa with DNA fragmentation was calculated per patient by the ratio between the number of sperm nuclei positive for TUNEL assay and the total number of explored spermatozoa. A sperm DNA fragmentation rate above the 5% threshold value was considered abnormal [4].

### 4.4. Ovarian Stimulation and Oocyte Collection

The ovarian stimulation protocols consisted of either the use of a long-acting gonadotropin-releasing hormone agonist (GnRHa) or an antagonist protocol followed by recombinant FSH (Puregon, MSD, Puteaux, France; Gonal-F, Merck Serono, Martillac, France), purified hMG (Menopur, Ferring, Gentilly, France) or purified urinary FSH (Fostimon, Juniper, Neuilly-sur-Seine, France). The dose of gonadotropins was adapted for each patient based on age, ovarian function and BMI. Monitoring was carried out before the start of gonadotropin injections to check for the absence of ovarian follicles larger than 10 mm after transvaginal ultrasound examination and/or E_2_ serum level greater than 50 pg/mL. The next monitoring took place on the sixth day of the attempt to adapt gonadotropin doses if necessary, according to E_2_ serum level correlated to the number and size of follicles, and then every two to three days. Oocyte retrieval was performed using transvaginal ultrasound-guided follicle aspiration 36 h after injection of human chorionic gonadotropin (hCG) (Ovitrelle, Merck Serono, France) when more than three follicles greater than 15 mm and E_2_ serum level greater than 150–200 pg/mL per follicle greater than 15 mm after ultrasound examination were observed. 

### 4.5. ICSI Procedure and Embryo Culture

Oocyte retrieval was performed in the operating room under general anesthesia, by transvaginal ultrasound-guided follicle aspiration. The oocyte–cumulus complexes were mechanically denudated with needles and enzymatically denudated using hyaluronidase (Syn vitro Hyadase, Origio, France). Sperm selection was performed after discontinuous density gradient using a 2-layer gradient of 70% and 90% fractions of Puresperm (PureSperm 100, JCD, La Mulatière, France) diluted in IVF medium (Origio, Limonest, France) and centrifugation at 1400 rpm for 20 min. The 90% fraction was washed with IVF medium (Origio, Limonest, France) by centrifugation at 2000 rpm for 10 min. The final pellet was resuspended in 0.5 mL of IVF medium (Origio) and incubated at 37 °C with 5% CO_2_. Then, spermatozoa were selected for ICSI in a drop of polyvinylpyrrolidone (PVP, Origio, France) before being immobilized by a flagellum lesion using an injection pipette (ICSI Micropipets, Origio, France). Only spermatozoa with progressive motility and normal form (sperm head, nucleus and flagellum) were selected at ×400 magnification under an inverted microscope (DMIRB1, Leica, France). Mature oocytes were injected using micromanipulators (Eppendorf, Germany) mounted on an inverted microscope within 2 h after oocyte retrieval, cultured in Global medium (LifeGlobal, JCD, France) supplemented with 10% HSA (LifeGlobal, JCD, France) under oil (Paraffin oil, Origio, France) and placed in the tri-gas time-lapse incubator (Embryoscope, Unisense Fertilitech, Aarhus, Denmark) at 37 °C with an atmosphere of 5% O_2_ and 6% CO_2_ for the duration of the culture. The camera was set up for the automatic recording of images of each embryo every 10 min, in seven focal planes. The images and related data were stored in the Embryoviewer (Unisense FertiliTech). The morphokinetic parameters observed were as follows: the time of the second polar body emission (tPB2), the time of appearance of two *pronuclei* (tPNa), the fading of both *pronuclei* (tPNf), the division to two blastomeres (t2), the division to three blastomeres (t3), the division to four blastomeres (t4), the duration of the first cell cycle (ECC1 = t2-tPB2), the duration of the second cell cycle (ECC2 = t3-t2), the time period to complete synchronous divisions (S2 = t4-t3) and the length of S-phase (tPNF-tPNa).

The choice of the embryo to be transferred was performed according to the morphologic and morphokinetic parameters of the embryos [44,45], the age of the female partner, and the medical history of the female and male partners. The classic morphological appearance of the embryos was assessed on day 2 based on the number and size of blastomeres (regular or irregular cleavage), the fragmentation rate (percentage of anucleate fragments) and the presence of multinucleated blastomeres [44]. Embryos with four regular blastomeres and less than 20% fragmentation were defined as “top-quality” embryos (type A). The number of embryos transferred depended on their morphology and morphokinetics as well as the age and medical antecedent of the female partner. After transfer, the luteal phase of the female partner was supported by vaginal progesterone (200 mg twice daily) and Provames (Sanofi-Aventis, France) for 14 days. The β-hCG plasmatic level was assessed 14 days after embryo transfer. An ongoing clinical pregnancy was defined by the visualization of an intrauterine gestational sac with an embryo presenting cardiac activity on ultrasound examination performed at 8 weeks of amenorrhea. The remaining embryos exhibiting good morphology and morphokinetic parameters were cryopreserved.

The fertilization rate (%) was estimated by the ratio between the number of fertilized oocytes and the number of microinjected mature oocytes. The cleavage rate (%) was estimated by the ratio between the number of embryos on day 2 (D2) and the number of fertilized oocytes with two pronuclei. The implantation rate (%) was estimated by the number of intrauterine gestational sacs per number of transferred embryos on D2. Embryos selected for transfer or cryopreservation based on morphology and morphokinetic parameters as well as discarded embryos were explored.

### 4.6. Outcome

Live birth was the main endpoint of the current study. We associated the features recorded in the early embryonic stages with the occurrence of live birth, the conventional semen parameters and the sperm nuclear damage (DNA fragmentation, chromatin condensation state).

### 4.7. Data Collection and Statistical Analysis

Clinical and biological characteristics of the patients were extracted from Medifirst software (Montigny le Bretonneux, France) and were expressed as mean and standard deviation for quantitative variables and as frequencies for categorical variables. Embryo morphokinetic parameters were extracted from the Embryoviewer (Unisense Fertilitech) and expressed as median and quartiles ([Q1; Q3]). All data were collected in an Excel spreadsheet and analyzed with GraphPad Prism (San Diego, CA, USA).

The main ICSI outcome parameter explored in our study was the occurrence of a clinical pregnancy. Patient characteristics, conventional semen parameters, sperm nuclear damage (DNA fragmentation, chromatin condensation state) and ICSI cycle parameters were described and compared depending on the main ICSI outcome and the sperm chromatin condensation state (NSCC vs. ASCC) using the Mann–Whitney test for quantitative and continuous variables or Fisher’s exact test for qualitative categorical variables. The Spearman test was used to evaluate the correlation between conventional semen parameters, sperm chromatin condensation state and morphokinetic parameters. The morphologic and morphokinetic parameters of the embryos were described and compared depending on the sperm chromatin condensation state (NSCC vs. ASCC) and live birth rate. A *p*-value of less than 0.05 was considered significant.

## Figures and Tables

**Table 1:** Demographic and clinical characteristics, conventional semen parameters and sperm nuclear damage (chromatin condensation defect and DNA fragmentation) of 157 infertile couples undergoing 157 ICSI cycles according to live birth outcome and sperm chromatin condensation state.

		Live Birth					
Characteristics	Couples *n* = 157 Mean ± SD or %	Yes *n* = 57 Mean ± SD or %	No *n* = 100 Mean ± SD or %	*p*	NSCC *n* = 136 Mean ± SD or %	ASCC *n* = 21 Mean ± SD or %	*p*
Women							
Delay of infertility (years)		4.2 ± 2.4	5.1 ± 3.2	0.06	4.6 ± 2.9	5.9 ± 3.4	0.1
Age (years)	33.7 ± 4.9	33 ± 4.6	34.1 ± 5	0.2	33.4 ± 4.6	35.1 ± 6	0.2
BMI (kg/m^2^)	24.1 ± 4.3	23.2 ± 4.2	24.7 ± 4.3	0.04	24.3 ± 4.3	23.1 ± 4.8	0.2
Basal serum FSH (UI/L)	6.6 ± 1.7	6.4 ± 1.6	6.7 ± 1.7	0.2	6.6 ± 1.7	7 ± 1.8	0.2
Basal serum LH (UI/L)	5.2 ± 2.4	5.1 ± 2.1	5.3 ± 2.6	0.6	5.3 ± 2.5	4.8 ± 2.1	0.4
AMH (ng/mL)	4.7 ± 4.1	5.6 ± 5.1	4.1 ± 3.3	0.02	4.9 ± 4.4	3.3 ± 1.9	0.1
Basal serum 17β-E2 (pg/mL)	42.2 ± 20.3	44.4 ± 24.2	40.8 ± 17.6	0.3	42.6 ± 21.1	40.4 ± 15.1	0.6
Men							
Age (years)	36.1 ± 5.9	35.5 ± 5.6	36.5 ± 6.1	0.3	35.9 ± 5.8	37.5 ± 6.5	0.2
BMI (kg/m^2^)	25.2 ± 4.7	24.3 ± 3.7	25.7 ± 5.2	0.1	25.3 ± 4.8	23.9 ± 3.5	0.3
Concentration (×10^6^/mL)	15.2 ± 20.2	14.7 ± 22.6	15.6 ± 18.7	0.8	15.6 ± 21.3	13 ± 11.1	0.7
Total sperm number (×10^6^/ejaculate)	50.2 ± 67.4	45 ± 71	49.4 ± 64.3	0.7	51.8 ± 7	40.3 ± 38.9	0.97
Sperm progressive motility (a + b, %)	26.4 ± 10.3	25.3 ±9.6	27.1 ±10.6	0.3	27 ± 9.7	22.5 ± 12.9	0.05
Normal sperm morphology (%)	26.8 ± 17.5	25 ± 18.6	28.1 ± 16.8	0.4	25.9 ± 17.7	31.7 ± 15.1	0.1
Abnormal chromatin condensation (%)	11.4 ± 7.3	11.8 ± 7.5	11.1 ± 7.2	0.5	9.1 ± 4.3	26.2 ± 4.7	<0.0001
DNA fragmentation (%)	7.9 ± 6.9	7.3 ± 6.2	8.2 ± 7.3	0.4	8.4 ± 10.4	9 ± 8.6	0.4

Continuous variables are presented as mean ± standard deviation. A *p*-value of <0.05 is considered significant. ASCC: abnormal sperm chromatin condensation; *n*: population size; NSCC: normal sperm chromatin condensation; sd: standard deviation.

**Table 2:** ICSI cycle parameters according to live birth outcome and sperm chromatin condensation state.

		Live Birth					
Parameters	Couples *n* = 157 Mean ± SD or %	Yes *n* = 57 Mean ± SD or %	No *n* = 100 Mean ± SD or %	*p*	NSCC *n* = 136 Mean ± SD or %	ASCC *n* = 21 Mean ± SD or %	*p*
Rank of ICSI cycle	1.6 ± 1.1	1.4 ± 0.9	1.7 ± 1.2	0.08	1.6 ± 1	1.8 ± 1.3	0.9
Total FSH administered doses (UI)	2109 ±1094	1832 ± 820	2284 ± 1208	0.01	2124 ± 1119	2009 ± 851.8	0.9
17β-E2 of hCG day (pg/mL)	2031 ± 803	1988 ± 788	2059 ± 815	0.6	2050 ± 837.6	1858 ± 509	0.3
Number of collected oocytes	11 ± 6.1	11.7 ± 6.1	10.5 ± 6.1	0.2	11.1 ± 6.3	9.6 ± 4.4	0.3
Number of injected oocytes	7.8 ± 4.6	8.2 ± 4.5	7.6 ± 4.6	0.4	7.9 ± 4.7	7 ± 3.6	0.5
Fertilization rate (%)	58.5 ±24.6	59.6 ± 23.9	57.8 ± 25.1	0.6	58 ± 24	57 ± 26	0.6
Number of embryos	4.4 ± 3.2	4.5 ± 3.1	4.3 ± 3.3	0.7	4.5 ± 3.2	3.7 ± 3	0.2
“Top”-quality embryos (%)	30.1 (205/681)	23.1 (64/276)	34.8 (141/405)	0.62	28.8 (174/603)	39.7 (31/78)	0.3
Number of transferred embryos per cycle	1.5 ± 0.6	1.6 ± 0.5	1.4 ± 0.6	0.02	1.5 ± 0.6	1.5 ± 0.6	0.9
Number of frozen embryos per cycle	1.55 ± 2.13	1.39 ± 1.89	1.65 ± 2.28	0.7	1.6 ± 2.2	1 ± 1.3	0.4
Number of usable embryos per cycle	3.05 ± 2.16	3.03 ± 1.94	3.06 ± 2.3	0.6	3.12 ± 2.26	2.57 ± 1.24	0.4
Number of discarded embryos per cycle	1.28 ± 1.81	1.49 ± 1.98	1.15 ± 1.68	0.1	1.3 ± 1.73	1.14 ± 2.28	0.1
Biochemical pregnancy rate (%)	49.6 (74/157)	-	-	-	46.3 (63/136)	52.4 (11/21)	0.5
Clinical pregnancy rate (%)	39.4 (62/157)	-	-	-	37.5 (51/136)	52.4 (11/21)	0.2
Clinical pregnancy loss (%)	10.8 (17/157)	-	-	-	11.8 (16/136)	4.7 (1/21)	0.9
Implantation rate (%)	26.3 (62/235)	-	-	-	24.1 (49/203)	40.6 (13/32)	0.2
Live birth (%)	36.3 (57/157)	-	-	-	33.8 (47/136)	47.6 (10/21)	0.2

Data are presented as *n* (%) for categorical variables and mean ± standard deviation for continuous variables. A *p*-value of <0.05 is considered significant. ASCC: abnormal sperm chromatin condensation; *n*: population size; NSCC: normal sperm chromatin condensation; sd: standard deviation.

**Table 3:** Morphokinetic parameters of 681 embryos obtained after ICSI according to the occurrence of live birth and sperm chromatin condensation state.

Parameters (Hours)	Total Cohort (*n* = 681 Embryos)	Live Birth		*p*	NSCC (*n* = 603)	ASCC (*n* = 78)	*p*
		Yes (*n* = 276)	No (*n* = 405)				
tPB2	3.3 [2.8; 3.9]	3 [3; 4]	3 [3; 4]	0.86	3.3 [2.8; 4]	3.3 [2.9; 3.6]	0.5
tPNa	7.2 [6.3; 8.2]	7.2 [6.4; 8.1]	7.3 [6.3; 17.2]	0.86	7.3 [6.3; 8.3]	6.8 [6.4; 7.6]	0.2
tPNf	23.6 [22.2; 25.3]	23.6 [22.2; 25.7]	23.6 [22.1; 25.3]	0.89	23.8 [22.4; 25.6]	22.6 [21.3; 23.7]	0.03
t2	26.2 [24.8; 28.1]	26.6 [24.6; 25]	26.1 [25; 27.8]	0.63	26.6 [25.2; 28.3]	25.3 [23.8; 26.5]	0.02
t3	36.7 [34.5; 39.2]	36.78 [34.4; 39]	36.72 [34.5; 39.3]	0.80	36.9 [34.5; 39.4]	35.3 [33.7; 36.8]	0.08
t4	38.2 [34.5; 39.2]	38.1 [35.6; 40.1]	38.2 [35.9; 40.4]	0.79	38.4 [36; 40.5]	36.2 [25.5; 38.1]	0.05
ECC1	22.8 [21.4; 24.3]	23 [21.2; 24.7]	22.7 [21.4; 24]	0.65	23.5 [21.5; 24.3]	22.2 [20.5; 22.8]	0.02
ECC2	11.7 [10.7; 12.9]	11.69 [10.8; 12.8]	11.8 [10.7; 23.2]	0.69	11.2 [1.7; 12.9]	11.7 [11.6; 12.4]	0.6
S2	1.2 [0.6; 2.5]	1.08 [0.6; 2.2]	1.24 [0.63; 2.8]	0.44	2.5 [0.6; 2.7]	1.8 [0.7; 1.2]	0.5
S-phase length	16.4 [15; 18]	16.32 [15; 18.51]	16.39 [15; 17.9]	0.82	16.8 [15.1; 18]	16.1 [14.4; 16.8]	<0.001

For continuous variables, median and quartiles 1 (Q1) and 3 (Q3) are presented. A *p*-value of <0.05 is considered significant. ASCC: abnormal sperm chromatin condensation; *n*: population size; NSCC: normal sperm chromatin condensation; sd: standard deviation.

## Data Availability

All data that support the findings of this study are available from the corresponding author upon request.

## Abbreviations

AB	Aniline blue
AFC	Antral follicle count
AMH	Anti-Müllerian hormone
ASCC	Abnormal sperm chromatin condensation
BMI	Body mass index
CECOS	Centre d’Etude et de Conservation des Œufs et du Sperme
D	Day
DNA	Desoxyribonucleic acid
E_2_	Estradiol
ECC1	Duration of the first cell cycle
ECC2	Duration of the second cell cycle
FSH	Follicle-stimulating hormone
GnRHa	Gonadotropin-releasing hormone agonist
hCG	Human chorionic gonadotrophin
HIV	Human immunodeficiency virus
ICSI	Intracytoplasmic sperm injection
IMSI	Intracytoplasmic morphologically selected sperm injection
IVF	In vitro fertilization
LH	Luteinizing hormone
n	Population size
NSCC	Normal sperm chromatin condensation
PBS	Phosphate-buffered saline
S2	Time period to complete synchronous divisions
SD	Standard deviation
t2	Time to two blastomeres
t3	Time to three blastomeres
t4	Time to four blastomeres
tPB2	Time of the second polar body expulsion
tPNa	Time of pronuclei appearance
tPNf	Time of pronuclei fading or disappearance
TUNEL	Terminal deoxynucleotidyl transferase dUTP nick-end labeling
WHO	World Health Organization
